# A Potential Role for the Ketogenic Diet in Alzheimer’s Disease Treatment: Exploring Pre-Clinical and Clinical Evidence

**DOI:** 10.3390/metabo14010025

**Published:** 2023-12-29

**Authors:** Tadeu P. D. Oliveira, Ana L. B. Morais, Pedro L. B. dos Reis, András Palotás, Luciene B. Vieira

**Affiliations:** 1 Departamento de Fisiologia e Centro de Investigação em Medicina Molecular (CIMUS), Universidad De Santiago de Compostela, 15782 Santiago de Compostela, Spain; tadeu.deoliveira.diz@usc.es; 2 Departamento de Farmacologia, Instituto de Ciências Biológicas (ICB), Universidade Federal de Minas Gerais, Belo Horizonte 31270-901, Brazil; analuisabatista@far.grad.ufmg.br (A.L.B.M.); pedrolucas0611@ufmg.br (P.L.B.d.R.); 3 Asklepios-Med (Private Medical Practice and Research Center), H-6722 Szeged, Hungary; palotas@asklepios-med.eu; 4 Kazan Federal University, Kazan R-420012, Russia; 5 Tokaj-Hegyalja University, H-3910 Tokaj, Hungary

**Keywords:** Alzheimer’s disease, ketogenic diet, ketone bodies, neurodegenerative diseases

## Abstract

Given the remarkable progress in global health and overall quality of life, the significant rise in life expectancy has become intertwined with the surging occurrence of neurodegenerative disorders (NDs). This emerging trend is poised to pose a substantial challenge to the fields of medicine and public health in the years ahead. In this context, Alzheimer’s disease (AD) is regarded as an ND that causes recent memory loss, motor impairment and cognitive deficits. AD is the most common cause of dementia in the elderly and its development is linked to multifactorial interactions between the environment, genetics, aging and lifestyle. The pathological hallmarks in AD are the accumulation of β-amyloid peptide (Aβ), the hyperphosphorylation of tau protein, neurotoxic events and impaired glucose metabolism. Due to pharmacological limitations and in view of the prevailing glycemic hypometabolism, the ketogenic diet (KD) emerges as a promising non-pharmacological possibility for managing AD, an approach that has already demonstrated efficacy in addressing other disorders, notably epilepsy. The KD consists of a food regimen in which carbohydrate intake is discouraged at the expense of increased lipid consumption, inducing metabolic ketosis whereby the main source of energy becomes ketone bodies instead of glucose. Thus, under these dietary conditions, neuronal death via lack of energy would be decreased, inasmuch as the metabolism of lipids is not impaired in AD. In this way, the clinical picture of patients with AD would potentially improve via the slowing down of symptoms and delaying of the progression of the disease. Hence, this review aims to explore the rationale behind utilizing the KD in AD treatment while emphasizing the metabolic interplay between the KD and the improvement of AD indicators, drawing insights from both preclinical and clinical investigations. Via a comprehensive examination of the studies detailed in this review, it is evident that the KD emerges as a promising alternative for managing AD. Moreover, its efficacy is notably enhanced when dietary composition is modified, thereby opening up innovative avenues for decreasing the progression of AD.

## 1. Review Design

The current narrative review was collaboratively designed by five contributors to plan the research question, drawing insights from considerations outlined in the abstract: “Ketogenic dietary therapies for neurodegenerative disease and Alzheimer’s disease”. Our research strategy was devised for PubMed and Scopus, aligning with predefined keywords. These keywords were employed both independently and in combination, utilizing the Boolean AND operator to establish logical relationships among concepts. The research approach involved advanced search techniques, and specific limits were set: papers published until July 2023 encompassing studies involving humans and rodent models, with a focus on English-language publications.

## 2. Epidemiology, Neuropathological Insights and Symptoms in Alzheimer ’s Disease

Alzheimer’s disease (AD) is the most prevalent form of dementia [1,2,3,4]. It is also considered to be a growing burden for the health care system and is one of the most expensive chronic diseases of old age [3,5,6,7]. In 2010, a total of 604 billion USD was spent on AD globally; these direct medical costs are exacerbated by lost productivity attributed to this debilitating disease. Projections for the year 2050 estimate that such costs would surpass 1 trillion USD in total [6,8,9,10]. These economic expenses are paralleled with the incidence of patients with Alzheimer’s, several of which are forecasted to rise above 150 million by 2050 [10,11]. In addition, AD has an intense impact on the individual and social spheres, with caregivers of affected people being more vulnerable to anxiety and depression [5,12]. Therefore, the functional capacity and autonomy of the elderly may be more important than mortality, as they relate to quality of life [6]. In all populations, AD is prevalent at advanced ages, notably between 70 and 80 years [2,13]. However, differences were found in the association of AD among ethnic groups and sex [14,15]. Importantly, a greater susceptibility to developing AD has been reported in Caucasians, Japanese, and women, as nearly two-thirds of people diagnosed are women [16,17].

Neuropathologically, AD is characterized by the deposition of β-amyloid peptides (Aβ) and the accumulation of neurofibrillary tangles (NFTs) composed of hyper-phosphorylated tau protein. Under physiological conditions, tau protein exists as a soluble and unfolded protein that interacts with tubulin to promote the assembly and stabilization of microtubules [18,19]. Aβ peptides can self-aggregate into soluble oligomers, or into insoluble fibers, forming the amyloid plaques. The Aβ from 36 to 43 amino acids is a product of proteolytic processing of protein amyloid precursor (APP), a type I transmembrane protein [20]. The cleavage of APP by the enzyme β-secretase (BACE-1) generates a C-terminal fragment (β-CTF), which is an immediate substrate for the γ-secretase enzyme that cleaves β-CTF to produce a spectrum of Aβ peptides with different lengths, i.e., the amyloidogenic pathway [21]. Conversely, when APP is cleaved by the α-secretase enzyme, it generates a C-terminus that subsequently undergoes cleavage by γ-secretase. This pathway, known as the non-amyloidogenic pathway, leads to the production of a shorter APP fragment (p3). This seemingly innocuous fragment may, in fact, hold pivotal roles in various processes, including cell growth, adhesion, synaptic plasticity, and the regulation of metal ion homeostasis [22,23].

Moreover, the amyloid hypothesis postulates that the accumulation of Aβ leads to a neurodegenerative cascade, resulting in synaptic dysfunction, NFT formation and, ultimately, neuronal loss in susceptible brain regions. Thus, AD is associated with neuronal degeneration, manifesting selective impairments in the hippocampus and neocortex regions, predominantly affecting cholinergic neuronal populations, and resulting in neurological impairments [24,25,26]. While the manifestation of symptoms may exhibit heterogeneity, AD is characterized by a progressive and irreversible decline in memory, cognition, thinking and language [27,28,29]. This decline culminates in diminished independence in performing daily tasks, accompanied by behavioral and motor impairments, as well as escalating psychiatric symptoms. These challenges tend to intensify as the neurodegenerative syndrome progresses [16,24,30]. Moreover, shared mechanisms of cell death in AD are related to increased oxidative stress, mitochondrial injury, hypometabolism, disruption of the blood–brain barrier (BBB) and neuroinflammation [31,32,33]. In the latter process, the increased formation of Aβ and NFTs is associated with the presence of activated microglia, cytokines, cyclooxygenase 2 (COX-2) and other inflammatory substances [28,32].

## 3. Risk Factors for AD

Despite 115 years having passed since Alois Alzheimer first described the disease, our understanding of AD remains incomplete. Consequently, researchers have explored genetic and environmental factors as potentially influential elements in the development of this condition [34,35]. Genetic risk factors for AD include mutations in amyloid precursor protein (APP), presenilin 1 (PSEN1), presenilin 2 (PSEN2) and apolipoprotein E4 (APOE4). Nonetheless, APOE4 stands as the most extensively established genetic risk factor for AD susceptibility beyond the age of 65 years [36,37,38,39]. It is linked to an elevated risk of NFT formation and a reduction in Aβ clearance, resulting in the accumulation of neurotoxic fragments [37,38]. Furthermore, while genes play a role in the risk of developing AD, they may account for only a modest portion of that risk [2,36]. It is important to highlight that the risk factors identified for AD share features and are not independent [40]. Notably, some of these factors encompass treatable medical conditions like stroke, hypertension and diabetes [36]. Epidemiologic studies revealed that the risk of AD is increased by 50–100% by type 2 diabetes mellitus (T2DM). A notable observation is that the APOE4 gene serves as a risk factor for the development of type 2 diabetes mellitus (T2DM), although it is important to recognize that not all individuals with T2DM possess the APOE4 gene. Additionally, this apolipoprotein has been linked to a reduction in glucose consumption by the brain [41]. Thus, T2DM is linked to an increased risk of developing late-onset AD (LOAD) [42,43]. Regarding modifiable environmental risks, numerous reviews have emphasized the substantial evidence associating smoking [42], nutrition [44] and obesity [45], and such findings demonstrate that behavioral aspects can influence the onset of clinical manifestations of AD [46,47,48].

## 4. Pharmacological Treatment for AD

Currently, there is neither a cure for AD [49] nor an effective drug for prevention or treatment that modifies the progression of the disease [41,50]. Given the severity of AD, advances in pharmacological treatments are progressing slowly [51], as only a few drugs are approved. The drug therapy for AD is symptomatic or palliative and not effective for advanced stages of the disease [49,52,53,54]. Such pharmacological treatments are focused on improving cholinergic transmission, and they are divided by the mechanism of action into two classes: cholinesterase inhibitors (ChEIs), used for mild to moderate stages, and an NMDA receptor antagonist (N-Methyl-D-aspartate), Memantine [49], used for moderate to severe stages or in cases of intolerance and contraindication [8,55,56]. ChEIs have moderate symptomatic benefits regarding cognition, functionality and behavior [36,57,58]. This class includes Donepezil, Galantamine and Rivastigmine [59]. Nevertheless, these drugs exhibit variations in certain pharmacological characteristics: Donepezil and Rivastigmine boast longer half-lives, but, in addition to acetylcholinesterase inhibition, Rivastigmine also deactivates butyrylcholinesterase. On the other hand, Galantamine demonstrates an additional enhancement in nicotinic receptor transmission [60,61]. As for the NMDA receptor antagonist, Memantine improves cognition, functionality and the management of agitation and aggression [36,58,62]. Furthermore, combining Memantine with Donepezil can lead to improved patient outcomes [61].

Additional treatment options are non-pharmacological, often more cost-effective and dependent on human effort [63]. This category encompasses numerous suggested approaches, including socialization [64], cognitive training [65], calorie restriction and exercise [8,60]. Among these alternatives, the ketogenic diet (KD) is currently under investigation as an adjuvant therapy [66,67,68,69].

## 5. Ketogenic Diet and Ketone Body Biosynthesis

**Figure 1:**
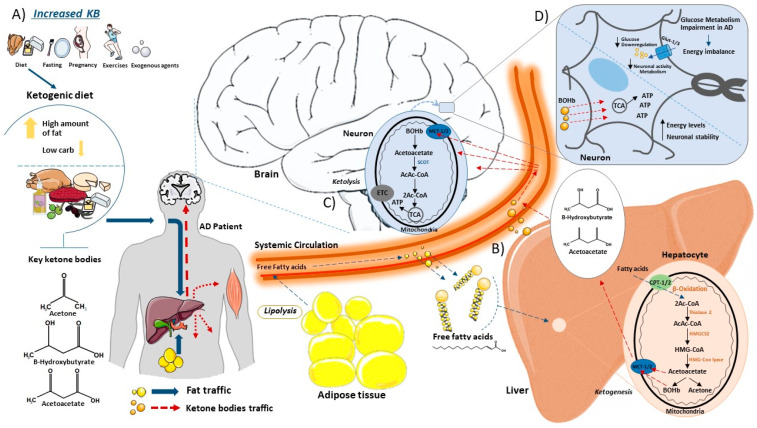
This schematic illustrates the synthesis and catabolism of ketone bodies within the body. (**A**) Ketones are produced in the liver under certain conditions. Consuming a high-fat low-carbohydrate diet promotes the production of ketone bodies as acetone, beta-hydroxybutyrate (BOHb) and acetoacetate in the liver. (**B**) Once in the bloodstream, free fatty acids from the ketogenic diet or adipocytes (lipolysis) enter the liver via the hepatic portal vein, where they participate in the ketogenesis process. Inside liver cells, free fatty acids access mitochondria via Carnitine Palmitoyltransferase (CPT) transporter channels. Within the mitochondria, fatty acids undergo oxidation, leading to the formation of essential ketone bodies. These ketone bodies exit the mitochondria through Monocarboxylate Transporter (MCT) channels, eventually leaving hepatocytes and entering the bloodstream, en route to the brain. (**C**) In the brain, ketone bodies enter the neuronal mitochondrial matrix through Monocarboxylate Transporters (MCT channels), where they undergo ketolysis. The action of Succinyl-CoA on ketone bodies promotes the formation of Acetyl-CoA, which subsequently integrates into the tricarboxylic acid (TCA) cycle for ATP generation. (**D**) Alzheimer’s disease is intricately linked to impaired glucose metabolism, resulting in an energy imbalance. Reduced glucose levels in cells compromise neuronal activity and metabolism. In this scenario, with diminished glucose availability, ketone bodies become the primary source for ATP production, allowing cells to maintain high energy levels and neuronal stability. This figure was partly generated using Servier Medical Art, provided by Servier, licensed under a Creative Commons Attribution 3.0 unported license.

The KD is a diet based on reducing carbohydrate consumption and increasing lipid intake [70,71]. This leads to a decrease in the use of glucose, which is no longer the main energy source, promoting the use of ketone bodies (ketones) from the breakdown of fatty acids (FAs) [72,73,74]. The hepatic metabolism of FAs produces ketones commonly used as substrates for energy: acetoacetate, acetone and beta-hydroxybutyrate (BOHb) [75,76]. Normally, ketones are produced in starvation [77], fasting [78,79], prolonged physical exercises [80], pregnancy [81] and in diets with high-fat and low-carbohydrate rates [82]. Thus, when glucose stores in the body are low, more FAs are made available to the liver for oxidation, leading to the consequent production of energy-rich molecules, mainly acetyl-CoA. Acetyl-CoA can enter the citric acid cycle in the liver or be used for the synthesis of ketones. Once in the liver cells, the fatty acid will be directed to the mitochondrial matrix by carnitine palmitoyltransferase (CPT1/2) where it initially undergoes a β-oxidation generating Acetyl-CoA that will undergo the process of ketogenesis [83]. In sequence in the process, the thiolase-2 enzyme acts in the conversion of two molecules of Acetyl-CoA to Acetoacetyl-CoA (AcAc-CoA) [83,84]. This molecule undergoes catalysis by the enzyme 3-Hydroxymethyl glutaryl-CoA synthase 2 (HMGCS2), resulting in the generation of hydroxymethylglutaryl-CoA (HMG-CoA) [84]. Subsequently, HMG-CoA is converted into acetoacetate and Acetyl-CoA via the catalytic action of HMG-CoA lyase [85]. Furthermore, acetoacetate can be reduced to D-β-hydroxybutyrate (D-βOHB) or decarboxylated to acetone [83]. After their formation, ketones are released from cells by monocarboxylate transporters (MCT1/2) and fall into the bloodstream to reach extrahepatic tissues for terminal oxidation [83]. Through the same MCT1/2 channels, ketone bodies enter the mitochondrial matrix of cells where they undergo the action of Succinyl-CoA: 3-ketoacid CoA transferase (SCOT) that transfers the CoA portion of succinyl-CoA to form Acetoacetyl-CoA [86]. The final part of the inverse process leads to the formation of Acetyl-CoA that is introduced into the TCA cycle for the formation of ATP that is used as an energy source in cases of glucose deprivation [87] (Figure 1).

## 6. Types of KD

There are different types of KDs, listed as: classic long-chain triglyceride KD (LCT), medium-chain triglyceride KD (MCT), modified Atkins diet (MAD) and low glycemic index diet (LOGI) (Table 1) [66,88,89,90]. The four diets have the same original formula, characterized by a high rate of fat and low amount of carbohydrate in their composition. However, they have occasional variations in the composition weight and ingredient restrictions [90]. LCT offers around 90% of energy in the form of fat and 10% of carbohydrates and proteins [90]. The most recommended ratio is 4:1 to 3:1 (fats: proteins and carbohydrates), but the use of each diet can be evaluated based on the patient’s profile and the most appropriate type of diet [90]. The diet ratio represents the balance between fat and protein plus carbohydrate grams. For instance, a “1800 kcal 4:1 ratio classic KD” contains four times the grams of fat compared to protein. This ratio can be customized to enhance seizure management or to make it more accommodating for improved tolerance. In contrast to the standard KD, the MCTKD is not influenced by food ratios; instead, it depends on the proportion of calories derived from MCT oil as a crucial source of ketones [91]. 

MCT has a distinct composition, primarily comprising about 60% octanoic acid, an eight-carbon fatty acid, and roughly 40% decanoic acid, a ten-carbon fatty acid [92]. Unlike the conventional KD, which relies more on medium-chain fats for dietary energy, the MCT-based diet permits a broader inclusion of carbohydrates. Due to the swift metabolism of these shorter fatty acids, this distinction results in a more efficient synthesis of ketones [92]. The process starts with dietary triglycerides in the form of MCT supplements, undergoing breakdown in the gastrointestinal tract by specialized lipases with a preference for hydrolyzing medium-chain esters rather than long-chain esters. Consequently, MCTs are converted into medium-chain fatty acids, characterized by their carbon atom content ranging from six to twelve [93]. This unique property enables direct absorption through the intestinal wall, leading to swift transportation to the liver. Once in the liver, the medium-chain fatty acids, including decanoic acid and octanoic acid, undergo rapid metabolism via a process known as β-oxidation [93]. The integration of an MCT-rich diet with an elevated carbohydrate intake sets this approach apart from the conventional KD, offering a balanced and efficient means of achieving ketosis while harnessing the advantages of medium-chain fats [92].

Due to the highly restrictive dietary regimen and concerning side effects associated with KDs, their implementation in pediatric patients is challenging. In this context, the modified Atkins diet (MAD) emerges as a more balanced and easily applicable alternative dietary therapy. The advantages of increased tolerance and sustainable treatment approach could potentially position MAD as the preferred choice. Unlike the classic KD, MAD shares similar food choices but eliminates the need for precise ingredient weighing. It also deviates from a strict ketogenic ratio and lacks restrictions on protein, fluid and calories. MAD is used to treat some metabolic paroxysmal movement disorders, such as those observed in glucose transporter type 1 deficiency syndrome (GLUT1DS) [88,89,90].

Another type of diet that combines the principles of the Mediterranean diet with the macronutrient composition of a KD is the Mediterranean Ketogenic Diet (MKD). Inspired by the heart-healthy Mediterranean diet, the MKD incorporates high consumption of fruits, vegetables, whole grains and healthy fats, notably olive oil. Simultaneously, it aligns with the principles of a KD by emphasizing low carbohydrate intake and promoting a state of ketosis. The MKD includes the use of olive oil as a primary source of fat, moderate consumption of fish and poultry, and limited intake of red meat and processed foods. The MKD encompasses several common variations, including the Very Low-Calorie Ketogenic Diet (VLCKD) which is characterized by a low carbohydrate content (<50 g/day), 1–1.5 g of protein/kg of ideal body weight, 15–30 g of fat/day and a daily intake of about 500–800 calories [94]. In addition, the High-Fat Ketogenic Diet (HFKD) is based on a higher proportion of daily calories sourced from fat, typically ranging between 75–80%. In contrast to the traditional KD, the HFKD allows for a modestly increased protein intake, contributing to a more flexible nutritional profile [94].

## 7. Possible Risks of KD

The benefits of ketones produced in the liver go beyond the energy supply of tissues such as the brain, skeletal muscle and heart [83]. Ketones antagonize inflammatory processes and oxidative stress [95,96], acting as signaling mediators [83]. Although the KD presents possible benefits to the organism [97], its use can also promote adverse effects such as headache, gastrointestinal pain, constipation, nausea, fatty diarrhea, fatigue, vomiting and other gastrointestinal problems [98,99,100]. These symptoms are usually associated with acute use of the KD as reported in studies with young and adult patients [99,100]. All the symptoms caused by the KD in the first few days are usually called “keto flu” [100]. It has been shown that the acute symptoms pass after a short period of time and patients who use the KD for more than one year can report different types of symptoms, as vitamin and mineral deficiencies, kidney stones, hyperuricemia, lethargy and infectious diseases, which can be harmful [99].

In a study involving obese patients, the impact of a KD was assessed over a 24-week period. The findings demonstrated that the KD treatment yielded positive outcomes, including significant weight loss and improvements in the patients’ lipid profiles, with no notable adverse effects reported [101]. However, it is crucial to emphasize that individuals with a genetic predisposition to cholesterol metabolism dysregulation may experience an exaggerated rise in cholesterol levels when adhering to a KD. Moreover, there remain certain uncertainties surrounding the prolonged use of this diet, primarily due to the limited evidence available for durations exceeding one year. These uncertainties encompass concerns related to cardiovascular risks and disruptions in lipid metabolism as well as impacts on hormone regulation, such as insulin [102,103]. Although there is evidence supporting KD effectiveness in specific contexts, such as obesity, diabetes, non-alcoholic fatty liver disease (NAFLD) and kidney disease, it is crucial to recognize that individual responses to the KD can vary significantly [102,103].

## 8. The Use of the KD in ND

Since the 1920s, the KD has been employed as a therapeutic approach for various neurological disorders, most notably in the management of drug-resistant refractory childhood epilepsy [92,104,105]. More recently, its potential role has been explored in the context of several neurodegenerative diseases, including AD [66] and PD [105,106], as well as in psychiatric diseases such as schizophrenia [107,108], depression [109,110] and Borderline Syndrome [111]. The success behind the use of the KD is linked to its action in common features shared by most CNS diseases, such as glucose hypometabolism, energetic deficits, imbalanced GABA and glutamate transmission, inflammation and oxidative stress [70,92]. Notably in some neurodegenerative diseases, patients present failures in the expression of glucose receptors GLUT1 and GLUT3, which may lead to glucose hypometabolism, contributing to the aggravation of diseases [92,112,113]. For instance, Glut1 deficiency syndrome (Glut1DS) causes a delay in glucose transport through the blood–brain barrier (BBB), leading to decreased cerebrospinal fluid glucose levels even in the presence of normal blood glucose [114]. In this context, the KD can enhance the provision of ketones at the expense of carbohydrates, potentially leading to an increased production of ATP, protecting neurons from energetic deficits. This is supported by a study that demonstrated a 22% increase in the expression of BOHb transporters, a key component of the KD, in patients with schizophrenia [113]. Moreover, the imbalance between GABA and glutamate represents a common feature contributing to the characteristic seizures observed in many neuropsychiatric disorders [70]. Notably, the Medium-Chain Triglyceride Ketogenic Diet (MCT KD) achieves seizure control via decanoic acid, which selectively inhibits AMPA receptors as demonstrated in preclinical models [31,92]. Furthermore, the anti-inflammatory properties of the KD are associated with its ability to shift microglial cells from a pro-inflammatory state to an anti-inflammatory state, offering promise in the management of mental illnesses [115]. Additionally, the KD, primarily via its ketone constituents, exerts control over oxidative stress by influencing various metabolic and signaling factors [70,92]. This multifaceted action underscores the broad spectrum of the KD’s therapeutic potential in treating various CNS diseases, thereby encouraging further exploration of its utility in conditions such as AD.

## 9. The Rationality of KD Use in AD

The progressive deposition of Aβ peptides and increased levels of hyperphosphorylated tau protein trigger neurodegeneration and impaired glucose metabolism [116]. Indeed, deficiencies of the GLUT1 receptor have been reported in AD, leading to impairment of glucose transport through the blood–brain barrier (BBB), causing hypometabolism of glucose in the CNS and consequently, starvation of neurons because of inefficient glycolysis [92,112,117]. Thus, an adjuvant therapy route to overcome the energy inefficiency of glucose in AD may act as an alternative fuel for brain metabolism [118,119]. In this case, the KD offers high levels of fat that, when metabolized in the liver, generate ketones which can supply the brain energetically and prevent neuronal death and synapse loss [41]. KD treatment may lead to neuroprotective effects by reducing Aβ damage to mitochondria, increasing ATP production, and reducing oxidative stress and glutamate toxicity [120]. Improvements in mitochondrial function have been attributed to biochemical changes resulting from the inhibition of glycolysis and increased KB formation [41]. Furthermore, KD treatment elevates ketone levels, and this increase exerts a neuroprotective effect on aging brain cells by diminishing the expression of inflammatory and apoptotic mediators [41,121,122].

Indeed, intriguing hypotheses propose a multifaceted impact of ketones on gene expression alterations and the modulation of cell signaling cascades. These processes, in turn, appear to regulate neuronal excitability, bolster antioxidant defenses and maintain the redox balance within cells [123,124]. In addition, there are suggestions that KD treatment may impact the deposition of Aβ or tau, slowing down the underlying disease process [92,125]. Moreover, the KD may act to reestablish the misbalance between the GABAergic and glutamatergic neurotransmission present in AD [126,127]. It has been reported that Aβ may increase AMPA currents, leading to glutamatergic hyperactivity, neurotoxicity and memory loss in AD [128]. In addition, the KD showed improvement in neuronal survival via the inhibition of AMPA receptors [31,92]. Another crucial aspect is that KD therapy contributes to the preservation of synaptic activity. This is achieved by enhancing the GABA/Glutamate ratio via elevated levels of Krebs cycle intermediates and by activating ATP-sensitive potassium channels via mitochondrial metabolism [129]. Hence, the implementation of the KD may attenuate neuronal hyperexcitability that has been described in preclinical and early clinical stages of AD [130,131].

Also, neuroinflammation, brain cell atrophy and apoptosis observed in AD may be related to the deleterious cellular effects of phosphate toxicity. It seems that hyperphosphorylated tau engages in the significant utilization of phosphate; consequently, a diminished dietary phosphorus intake emerges as a potential strategy to mitigate the risk of AD [46]. Interestingly, some studies suggest that the KD may increase the excretion of phosphorus in urine. This could potentially lead to lower phosphate levels in the body [46].

## 10. KD Results in Preclinical Models of AD

Studies with preclinical models demonstrate that the KD can provide benefits regarding inflammation [130,132,133], pain [134,135], cancer [136], metabolic disorders [137,138,139], epilepsy [140,141], aging [142], memory [143,144], mood [145,146] and neurodegenerative diseases [147,148,149]. Within this latter group, the benefits of the KD in preclinical models of AD involve changes at molecular and cellular levels, culminating in symptom improvement [125,130] (Table 2).

One potential mechanism by which the KD operates in mouse models of AD is via the direct reduction of Aβ. An intriguing study utilizing the APP mouse model (APP/V717I) revealed that after 43 days of KD treatment, there was an approximate 25% decrease in brain Aβ levels (Aβ40 and Aβ42) [131]. Nevertheless, it is worth noting that in this model, no significant memory improvement was observed when assessed using the object recognition test [131]. In a preclinical study, 5XFAD mice, exposed to a KD for an extended duration of 4 months, exhibited notable cognitive improvements alongside reduced neurotoxicity and diminished neuroinflammation [130]. Moreover, a significant decrease in the accumulation of Aβ plaques was observed in the immunohistochemical analysis of the hippocampus of treated KD mice [130]. This is an important result as the accumulation of Aβ favors neurotoxic events in the brain [150,151]. Of note, following an evaluation of spatial learning, mice subjected to a KD displayed enhanced efficiency with shorter latency times in comparison to standard-diet-treated mice. Moreover, apart from the behavioral improvements, the KD-treated mice also exhibited significant preservation of synaptic density, along with reductions in neuroinflammation and microgliosis [130].

Curiously, some studies have demonstrated an improvement in AD phenotype without changes in the deposition of Aβ in the brain. An investigation employing different AD models, including Aβ deposition (APP/PS1) and a tau protein accumulation model (Tg4510), underscores the advantageous effects of the KD [125]. In this study, five-month-old animals were subjected to a KD for duration of 3 months. Notably, upon analyzing the deposition of tau and Aβ in the cortical and hippocampal brain regions, no discernible differences were observed between the KD-treated mice and those on a control diet [125]. However, KD-treated mice exhibited improved motor performance in the rotarod test [125].

Another interesting fact about the KD is the direct effect on neuroinflammation, a hallmark of AD [152,153]. The anti-inflammatory effect of the KD in preclinical models has been studied [134,135,154]. This potential anti-inflammatory effect may be linked to a direct action on the mTOR pathway [155,156], PPARγ [157,158], NLRP3 inflammasome [159], in addition to other targets such as ketone bodies [160], adenosine [141,161] and the intestinal microbiota [162]. Mice fed with a KD for duration of 4 weeks exhibited a notable reduction in neuroinflammation induced by the administration of kainic acid (KA). This suggests that the diet’s anti-inflammatory effects are closely linked to the activation of PPARγ activation [133]. Consequently, the KD, via PPARγ activation, led to a decrease in TNF-α and NF-kB levels in the hippocampus following KA administration. Furthermore, the KD effectively inhibited the expression of cyclooxygenase (COX-2) and microsomal prostaglandin E2 synthase-1 (mPGES-1) in the hippocampus. These findings suggest that the KD can attenuate neuroinflammation by suppressing the COX-2 dependent pathway via PPARγ activation [133]. It is worth noting that the literature indicates that increasing PPARγ expression in certain brain regions may reduce and even prevent the neuroinflammatory response [133,163]. This is attributed to the pivotal role of PPARγ in regulating pro-inflammatory cytokines [164], modulating microglia [165], regulating macrophage activation [166] and inhibiting transcription factors [167]. 

In obese rats subjected to a KD, a notable reduction in the expression of APP and apoE mRNA has been observed [168]. Furthermore, KD treatment resulted in weight loss, lowered cholesterol and glucose levels, and improved insulin sensitivity, alongside a reduction in inflammatory markers. These findings may provide insight into the KD’s favorable effects on APP expression in the brain [168]. Additionally, aside from impacting Aβ and tau deposition and neuroinflammation, the KD appears to induce morphological changes in microglial and astroglial cells [169]. 

Another noteworthy factor is the impact of the KD on the intestinal microbiota [73,170,171]. Notably, in a study involving young healthy mice (12–14 weeks old) subjected to chronic KD treatment, several significant effects were observed. These included an increase in peripheral plasma ketone levels, which corresponded with enhanced diversity and the presence of beneficial gut microbiota. At the central level, the study revealed an augmentation in cerebral blood flow and alterations in the expression of various proteins, including mTOR, P-glycoprotein transports and eNOS [172]. These results demonstrate an improvement in neurovascular function resulting from the decrease in mTOR activity and eNOS activation [172]. These findings suggest that changes in the microbiota may lead to protein alterations that enhance neurovascular activity, thereby mitigating the risk of AD [172]. 

Apart from the classic or standard KD, there is evidence that various KD variations can also yield positive results against AD in animal models [173,174]. In one study, the authors examined the effects of an 8-week treatment with medium-chain fatty acids derived from MCTs, namely MCT8, MCT10 or supplementation with sunflower oil (5% by weight of the chow diet) in 21-month-old Wistar rats. The 8-week MCT diets exhibited cognitive and physiological improvements in the rats. MCT treatment led to an increase in the expression of growth factors in the hippocampus and promoted alterations in synaptic markers, transcription factors, protein synthesis and plasticity [174]. In addition, treatment with MCT10, but not MCT8, significantly improved novel object recognition memory as compared to a control diet, while social recognition was increased in both MCT groups [174]. Also, treatment with supplements capable of inducing ketogenesis has shown promising results [120,175,176]. Ketogenic agents are an alternative to the use of a diet capable of producing the ketone bodies necessary for energy metabolism in cells [177]. Some exogenous agents, such as 2-deoxy-D-glucose (2-DG) and the ester of D-β-hydroxybutyrate and R-1,3 butane diol, referred to as ketone ester (KE), demonstrate a beneficial effect in a model of triple-transgenic AD mice (3xTgAD) [120,175,176]. 2-deoxy-D-glucose (2-DG), when administered over a 7-week period to 6-month-old 3xTgAD mice, led to an elevation in serum ketone body levels, subsequently resulting in a reduction in the pathological effects of AD in these mice [120]. The application of 2-deoxy-D-glucose (2-DG) was associated with a decrease in Aβ load, an improvement in mitochondrial bioenergetic capacity and an increase in the expression of neurotrophic growth factors. Conversely, the ketogenic ester (KE), when incorporated into the mice’s diet, contributed to increased ketone body levels, consequently diminishing protein and lipid oxidation and enhancing ATP hydrolysis energy [175]. A reduction in Aβ and hyperphosphorylated tau deposition levels was also noted. Surprisingly, these effects appeared to influence the behavior of these animals. The 3xTgAD mice exhibited decreased anxious behavior and performed better on tests of learning and memory [176]. These results underscore the positive effects resulting from the induction of ketone bodies via exogenous ketogenic agents.

**Table 2:** KD results in preclinical models of AD.

Author	Species Studied	Experimental Model	Diet/ Supplementation	Duration	Effects
Yao, J. et al. [120]	Mice (♀)	3xTgAD	2-deoxy-D-glucose (2-DG)	7 weeks	Reduced β-amyloid generation, increased β-amyloid clearance. Enhanced mitochondrial bioenergetic capacity and increased the expression of neurotrophic growth factors.
Brownlow, M. L. et al. [125]	Mice	APP/PS1-Tg4510	Medium-chain triglyceride KD	16 weeks	Improved motor performance in rotarod test.
Xu, Y. et al. [130]	Mice (♂)	5XFAD	Classic KD	4 months	Improved spatial learning, spatial memory and working memory. Restored number of neurons and synapses. Reduced neuroinflammation, amyloid plaque deposition and microglial activation.
Van der Auwera, I. et al. [131]	Mice (♀)	APP/V717I	Classic KD	38 days	Reduced levels of Aβ in brain tissue.
Liu, H. et al. [155]	Rats (♂)	Sprague-Dawley	KD with or without medium-chain fatty acids	30 days	Effects on the mTOR pathway and anti-inflammation action.
Mohamed, H. E. et al. [168]	Rats (♂)	Obesity induced with HFD	Classic KD	6 weeks	Improvement of brain oxidative stress responses. Downregulation of brain amyloid protein precursor, apolipoprotein E and caspase-3 mRNA expression.
Gzielo, K. et al. [169].	Rats (♂)	Wistar	Classic KD	4 months	Morphologically changes in microglial and astroglial cells.
Ma, D. et al. [172]	Mice (♂)	C57Bl/6	Classic KD	16 weeks	Increased CBF and P-glycoprotein transports on BBB. Reduced mTOR and increased eNOS protein expressions. Enhanced neurovascular functions. Increased the abundance of beneficial gut microbiota.
Wang, D. & Mitchell, E. S. [174]	Rats (♂)	Wistar	Medium-chain triglyceride KD	8 weeks	Increased expression of growth factors, alteration of synaptic markers, transcription factor, protein synthesis and plasticity. Cognitive improvement and difference performance in object and social recognition tests.
Pawlosky, R. J. et al. [175]	Mice (♂)	3xTgAD	Ketone ester (KE)	8 months	Corrected energy deficiencies in the hippocampus, improved biomarkers and reduced oxidative damage.
Kashiwaya, Y. et al. [176]	Mice (♂)	3xTgAD	Ketone ester (KE)	8 months	Improved behavioral cognitive function and decreased. Aβ and pTau pathologic changes.

Abbreviations: BBB = blood–brain barrier; CBF = cerebral blood flow; eNos = endothelial nitric oxide synthase. ♀ female and ♂ male.

## 11. KD Results in Clinical Trials

In addition to data acquired from preclinical research, several clinical studies demonstrate the feasibility and effectiveness of the KD in patients diagnosed with AD [94,178,179] (Table 3). In a randomized trial aimed at investigating the impact of a KD on patients with consistent AD diagnoses, those treated with a 12-week KD reported an improved quality of life and enhanced daily functionality [180]. Furthermore, the diet was found to be safe, with only mild adverse effects. Notably, it was observed that high levels of dietary lipids did not appear to pose significant cardiovascular risks for the patients [180]. It is worth mentioning that the sample size and duration of this trial are limiting factors. Specifically, a modified MKD, when provided to AD patients, is shown to enhance cognitive status while positively impacting metabolic and biomarker parameters [181]. Comparative analysis between the modified KD and a control group revealed improvements in cerebrospinal fluid biomarkers, including an increase in Aβ42 and a decrease in tau levels [181]. Other favorable outcomes encompassed increased uptake of brain ketone bodies, enhanced brain perfusion and improved peripheral lipid and glucose metabolism. Cognitive performance, as evaluated using the Free and Cued Selective Reminding Test (FCSRT), also exhibited notable improvements among patients [181].

Furthermore, it is important to note that studies have demonstrated the Mediterranean–KD’s modulating effect on the gut microbiome, both in patients with mild cognitive impairment [182] and in preclinical models [182]. A randomized, double-blind, cross-over pilot study conducted at a single center involved 17 subjects of whom 11 had mild cognitive impairment, while the remaining 6 displayed normal cognitive function. The study encompassed a 6-week intervention of the Modified Mediterranean–Ketogenic Diet (MMKD) and the American Heart Association Diet (AHAD) separated by 6-week washout periods. Assessments were made for gut microbiome composition, fecal short-chain fatty acids (SCFAs) and AD markers in cerebrospinal fluid (CSF), which included Aβ-40, Aβ-42, total tau and phosphorylated tau-181 (tau-p181), both before and after the dietary interventions. Interesting, the results of this study indicate that distinct gut microbial signatures could potentially serve as indicators of mild cognitive impairment, and further reveal that the MMKD can effectively reshape the gut microbiome and associated metabolites, correlating with improvements in AD biomarkers in cerebrospinal fluid (CSF).

The MCT diet applied to patients with AD also shows positive data regarding the pathological conditions of the disease [93,183,184]. Patients with mild to moderate AD sequentially ingested two distinct MCT supplements, each for a duration of one month: a blend of caprylic acid (55%) and capric acid (35%) (*n* = 11), followed by a wash-out period, and subsequently tricaprylin (95%; *n* = 6). Using PET imaging, the study quantified brain ketone (11C-acetoacetate) and glucose (FDG) uptake before and after each MCT intervention. As a result, the dietary treatment significantly enhanced overall brain energy metabolism in individuals with mild to moderate AD, while not impacting brain glucose utilization [93]. 

The MCT diet was also shown to be effective in producing ketosis and patients with AD demonstrated an overall cognitive improvement [183]. An interesting pilot study showed the KD Retention and feasibility trial involved a 3-month medium-chain triglyceride (MCT)-supplemented KD, followed by a 1-month washout period, among participants with clinical dementia ratings (CDR) of 0.5, 1 and 2. Throughout the study, data on urine acetoacetate, serum β-hydroxybutyrate, food intake records and safety were collected. The primary outcome of the study revealed that participants experienced an average improvement of 4.1 points in their scores on the Alzheimer’s Disease Assessment Scale-cognitive subscale (ADAS-Cog) from the beginning of the dietary intervention to its conclusion. However, these improvements on the ADAS-Cog waned after a one-month diet washout period [183]. 

Remarkably, noteworthy improvements were observed in short-term memory and processing speed among patients who underwent MCT diet treatment for several weeks [184]. A study involved 20 Japanese patients with mild-to-moderate AD, comprising eleven males and nine females. On separate days, these patients underwent neurocognitive tests 120 min after consuming 50 g of a ketogenic formula known as Ketonformula^®^, which contained 20 g of medium-chain triglycerides (MCTs). On alternate days, they consumed an isocaloric placebo formula lacking MCTs. Subsequently, the patients consumed ketogenic formula daily for up to 12 weeks and underwent monthly neurocognitive assessments. In the initial trial, following a single intake of the ketogenic formula, the patients exhibited a successful elevation in plasma levels of ketone bodies. However, there were no significant differences in cognitive test results between the administration of the ketogenic and placebo formulas. In the subsequent chronic intake trial of the ketogenic formula, 16 out of the 20 patients completed the 12-week regimen. After 8 weeks, patients showed significant improvement in their immediate and delayed logical memory test scores compared to their baseline scores. By the end of the 12-week period, they demonstrated significant improvements in the digit-symbol coding test and immediate logical memory test scores relative to their baseline [184]. 

A randomized placebo-controlled trial included six participants with mild cognitive impairment (MCI). This study evaluated the effect of the daily consumption of oil composed of medium-chain triglycerides (MCTs) for 24 weeks on serum ketone body concentrations, BOHb, apolipoprotein-E4 status and cognitive performance assessed using the Alzheimer’s disease Assessment Scale-Cognitive subscale (ADAS-Cog). The intake of MCT oil resulted in elevated concentrations of BOHb and demonstrated an enhancement in memory among subjects with MCI. It is important to note that the study had limitations, particularly the small sample size [185].

Another factor that gives more reliability and credibility to the use of the KD in the treatment of AD is the use of ketogenic agents in patients with mild to moderate conditions [186]. An oral ketogenic compound, AC-1202, was tested in subjects with probable AD to examine if ketosis could improve cognitive performance. AC-1202 rapidly elevated serum ketone bodies in AD patients and resulted in significant differences in ADAS-Cog scores compared to the placebo. The most prominent effects were observed in subjects without the APOE4 allele who adhered to the prescribed dosage regimen [187]. Furthermore, the daily consumption of caprylidene, a ketogenic compound that, upon metabolism, generates the ketones BOHb and acetoacetate, known for their ability to cross the BBB, resulted in increased blood flow within distinct brain regions among patients who lacked an apolipoprotein ε4 allele over a period of 45 days [188]. Moreover, the supplementation of MCT-based beverages has demonstrated cognitive enhancements in these patients [189,190]. Specifically, a daily intake of 30 g of ketogenic medium-chain triglycerides (kMCTs) over a span of 6 months generated sufficient ketones to notably improve brain energy levels in individuals with MCI. Notably, various aspects of cognitive function exhibited improvement in direct correlation with the enhanced brain energy levels achieved via kMCT supplementation. Future investigations will determine whether a larger sample size can validate the cognitive benefits of this kMCT dosage or if a higher dose is necessary. Nonetheless, the study’s authors underscored the feasibility of conducting long-term clinical trials with kMCTs and energy-equivalent placebos among older individuals. Subsequently, further research aimed at postponing age-related cognitive decline via the optimization of brain energy support with ketones is strongly justified [190].

## 12. Conclusions

This review aims to shed light on both preclinical and clinical studies exploring the potential of KD as an adjuvant therapy for AD. Given the absence of specific drugs or direct treatments targeting this neurological disorder, the most promising path for enhancing the quality of life for patients involves a synergistic approach, combining pharmaceutical interventions with complementary non-pharmacological strategies. Considering the energy metabolism deficits in neurons associated with AD, it appears reasonable to explore alternative approaches in combination with pharmacological treatments. By employing these complementary strategies alongside medications, we can potentially create a more resilient response within the body, thereby enhancing its ability to resist the progression of this disorder. Based on the analysis conducted in this study, it is recommended to conduct additional investigations within both preclinical and clinical models. This is essential to gain a deeper understanding of the effects of KD and to obtain more comprehensive data regarding the mechanisms via which this diet operates. While some animal models, such as those with APP mutations, have yielded promising results, a more comprehensive assessment encompassing various types of KD and diverse rodent models of Alzheimer’s disease is crucial. This comprehensive approach is vital to identify and elucidate the primary cellular pathways responsible for the beneficial effects of the diet in animals with AD pathology. For this, the use of knockout animals or those with the overexpression of key proteins linked to neuroprotective effects in the brain as PPARγ and NLRP3 can provide more concrete results. Simultaneously, there is a persistent requirement to enhance clinical studies by involving extensive patient cohorts, implementing more frequent follow-ups, refining cognitive assessment tests and refining sample collection methodologies for molecular analyses. Moreover, it is essential to conduct long-term studies to ascertain whether the favorable outcomes associated with the adoption of this diet can be consistently maintained over an extended period. In alignment with this, KD emerges as a promising adjuvant therapy option, although further clarification of its precise mechanism of action and potential side effects remains necessary. This study has lightened potential benefits of KD across both preclinical models and clinical research. Overall, the findings suggest that KD holds intriguing prospects for further enhancement and utilization in AD management. The different variations of this diet, along with dietary supplements inspired by its principles, may offer novel avenues for facing the progression of AD, potentially leading to innovative strategies for intervention.

## Figures and Tables

**Table 1:** Types of ketogenic diets.

KD Type	Ratio	Carbohydrate Intake per Day on a Diet of 1000 Kcal	Considerations
LCT	4:1 to 3:1	8 g on a 4:1 16 g on a 3:1	Severe carbohydrate restriction, unpalatable
MCTD	Not diet-ratio-related	48 g	More ketogenic, gastrointestinal side effects
MAD	Approximately 1:1	40–60 g	No precise weighing, no protein/calorie restrictions
LOGI	Approximately 1:1	10 g for the first month then 20–30 g	Minimized glycemic increases, liberalized regimen

Abbreviations: classic long-chain triglyceride KD (LCT), medium-chain triglyceride KD (MCT), modified Atkins diet (MAD) and low glycemic index diet (LOGI).

**Table 3:** KD results in clinical trials of AD.

Author	Participants (*n*)	Primary Diagnosis	Diet/ Supplementation	Duration	Ketosis	Results/Side Effects
Croteau, E. et al. [93]	15	Mild-moderate AD	Medium-chain triglyceride KD Suppl. C8C10 and C8	Two periods of 1 month	C8C10: Blood BOHb (mM) = 0.46 ± 0.19 C8: Blood BOHb (mM) = 0.57 ± 0.27	Increased total brain energy metabolism.
Phillips, M. C. L. et al. [180]	26	Alzheimer’s disease	Classic KD	Two periods of 12 weeks	BOHb level = 0.95 ± 0.34 mmol/L 18/21 patients	Improved daily function and quality of life. No significant changes were observed in the lipid profile. Mild adverse effects.
Neth, B. J. et al. [181]	20	Subjective memory complaints or mild cognitive impairment	Mediterranean KD	Two periods of 6 weeks	BOHb level = 0.23 (0.27) mmol/L	Improvement of peripheral metabolic measures, CSF biomarker profile and increased cerebral perfusion. No serious adverse events occurred.
Nagpal, R. et al. [182]	17	Mild cognitive impairment or cognitively normal	Mediterranean KD	Two periods of 6 weeks	Not measured	Modulating capacity of MMKD in the gut microbiome.
Taylor, M. K. et al. [183]	15	Very-mild, mild and moderate AD	Medium-chain triglyceride KD	3 months	Serum BOHb level = 0.31 mmol/L	Overall cognitive improvement. No serious adverse events occurred.
Ota, M. et al. [184]	20	Mild-moderate AD	Medium-chain triglyceride KD	12 months	Plasma BOHb level = 470.9 ± 292.6 μmol/L	Positive effects on verbal memory and processing speed. Diarrhea, most frequently reported side effect of MCT.
Rebello, C. J. et al. [185]	6	Mild cognitive impairment	Medium-chain triglyceride KD	24 weeks	ApoE4(−) Serum BOHb level = 0.15 mM ApoE4(+) Serum BOHb level = 0.54 mM	Memory improvement.
Ohnuma, T. et al. [186]	22	Sporadic, mild-moderate AD	AXONA Dietary Suplment	3 months	Serum BOHb level = 81.1 ± 79.9 μM	Improvement in cognitive functions. No severe gastrointestinal adverse effects.
Henderson, S. T. et al. [187]	152	Mild-moderate AD	AC-1202	90 days	BOHb level = 0.39 mM	Cognitive improvement. Mild to moderate adverse events restricted to the gastrointestinal system.
Torosyan, N. et al. [188]	16	Mild-moderate AD	Caprylidene	45 days	Not measured	Increased blood flow in specific brain regions.
Reger, M. A. et al. [189]	20	AD or mild cognitive	Emulsified medium-chain triglyceride	Two times of 90 min	ApoE4(−) Serum BOHb level = 0.54 mM ApoE4(+) Serum BOHb level = 0.43 mM	Cognitive improvement in AD patients withoutAPoE4.
Fortier, M. et al. [190]	52	Mild cognitive impairment	Ketogenic medium-chain triglyceride drink	6 months	Plasma BOHb level = 401 ± 303 μM	Improved several cognitive outcomes in MCI. No severe adverse events.

Abbreviations: ApoE4 = apolipoprotein E4; BOHb = beta-hydroxybutyrate; CSF = cerebral spinal fluid. +: plus APOE4; −: without APOE4.
